# Supplementary material for: The Active Site of the Enzyme 10-Formyl-THFDH in the Honey Bee Apis mellifera—A Key Player in Formic Acid Detoxification
Source: Int J Mol Sci. 2022 Dec 26;24(1):354. doi: 10.3390/ijms24010354 (PMC9820478; doi:10.3390/ijms24010354)
Supplement: Supplementary file 1 [file ijms-24-00354-s001.zip › ijms-2093807-supplementary.pdf]

International Journal of
Molecular Sciences

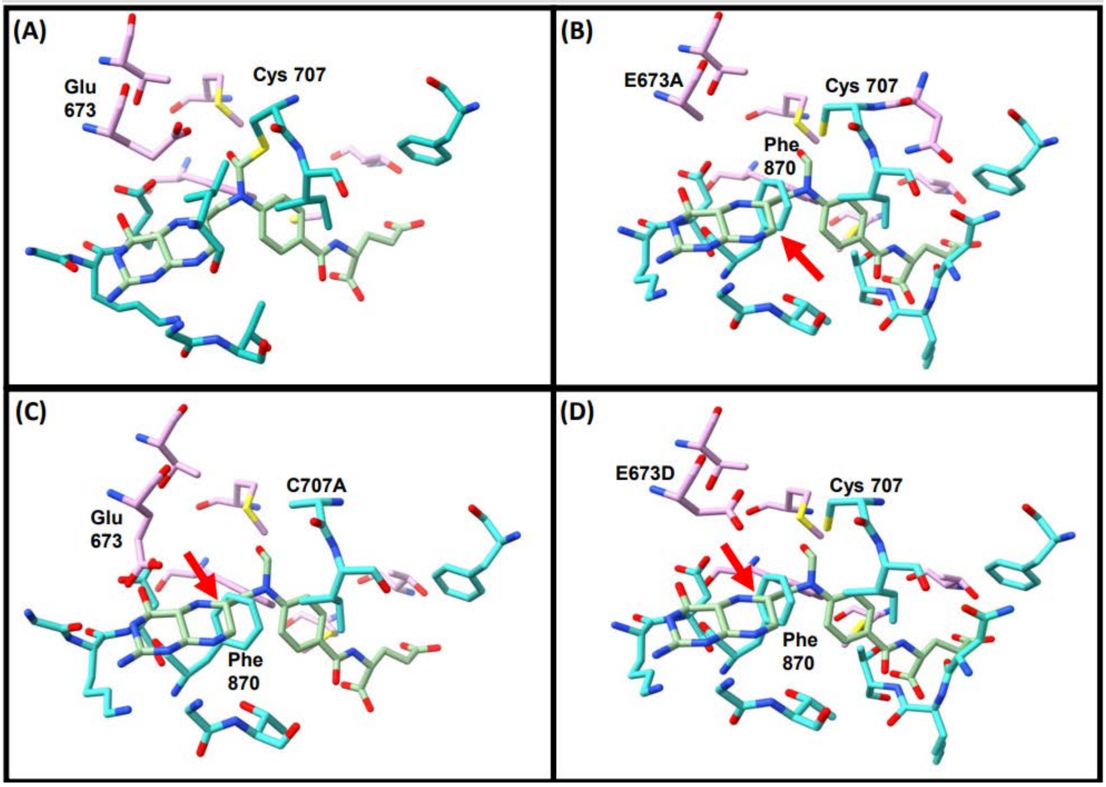

Figure S1 Docking of substrate to mutants. This figure shows the different docking results for the 3 mutants and the wildtype enzyme. (A) shows the wildtype enzyme with its substrate bound. It is shown, that the C707 can covalently bind to the substrate. (B) shows the mutation of C707A, where no covalent binding can occur between the alanine and the substrate, additionally a sterical clash between a phenylalanine (F870) of the enzyme and the substrate occurs (red arrow). (C) and (D) show the mutation of E673 to D and A, respectively, where again a sterical clash occurs between the phenylalanine (F870) of the enzyme and the substrate (red arrow).

```
Homo_sapiens                          KSCAISNVKKVSLELGGKSPLIIFADCDLNKAVQMGMSSVFFNKGENCIAAGRLFVEDSI
Pongo_abelii                          KSCAISNVKKVSLELGGKSPLIIFADCDLNKAVQMGMSSVFFNKGENCIAAGRLFVEDSI
Rattus_norvegicus                     KSCALSNVKKVSLELGGKSPLIIFADCDLNKAVQMGMSSVFFNKGENCIAAGRLFVEESI
Mus_musculus                          KSCALSNVKKVSLELGGKSPLIIFADCDLNKAVQMGMSSVFFNKGENCIAAGRLFVEDSI
Bombus_terrestris                     SCCAKSNLKKVSLELGGKSPLVIFEDTDLQQAVKIGMSSVFFNKGENCIAAGRLFVEETI
Bombus_pyrosoma                       RCCANSNLKKVSLELGGKSPLVIFEDTDLQQAVKIGMSSVFFNKGENCIAAGRLFVEETI
Bombus_impatiens                      RCCANSNLKKVSLELGGKSPLVIFEDTDLQQAVKIGMSSVFFNKGENCIAAGRLFVEETI
Bombus_huntii                         RCCANSNLKKVSLELGGKSPLVIFEDTDLQQAVKIGMSSVFFNKGENCIAAGRLFVEETI
Bombus_vancouverensis_nearcticus      RCCANSNLKKVSLELGGKSPLVIFEDTDLQQAVKIGMSSVFFNKGENCIAAGRLFVEETI
Bombus_bifarius                       RCCANSNLKKVSLELGGKSPLVIFEDTDLQQAVKIGMSSVFFNKGENCIAAGRLFVEETI
Bombus_vosnesenskii                   RCCANSNLKKVSLELGGKSPLVIFEDTDLQQAVKIGMSSVFFNKGENCIAAGRLFVEETI
Eufriesea_mexicana                    RSCADSNLKKVSLELGGKSPLVIFEDADLQQAVKVGLNSVFFNKGENCIAAGRIFVEETI
Apis_florea                           RSCANSNLKKVSLELGGKSPLVIFEDADLQQAIKIAISSVFFNKGENCIAAGRLFVEETI
Apis_dorsata                          KSCANSNLKKVSLELGGKSPLVIFEDTDLQQAIKIAMSSVFFNKGENCIAAGRLFVEETI
Apis_laboriosa                        KSCANSNLKKVSLELGGKSPLVIFEDTDLQQAIKIAMSSVFFNKGENCIAAGRLFVEETI
Apis_cerana                           KSCANSNLKKVSLELGGKSPLVIFEDTDLQQAIKIAMNSVFFNKGENCIAAGRLFVEETI
Apis_mellifera                        KSCANSNLKKVSLELGGKSPLVIFEDTDLQQAIKIAMSSVFFNKGENCIAAGRLFVEETI
                                       .** **:*************:** * **::*:::.:.***************:***::*
```

Figure S2 This figure shows the multiple sequence alignment of the amino acid sequence of the enzyme 10-formyl-THFDH of different species of the *Apidae* family and *Mammalia*. Highlighted in purple are the identified important residues E673 and C707. The whole active region is highly conserved within the family ("*" indicates complete conservation of amino acids; ":" indicates few exchanges with same functional group of amino acids; "." Indicates general conservation with few exchanges of amino acids.

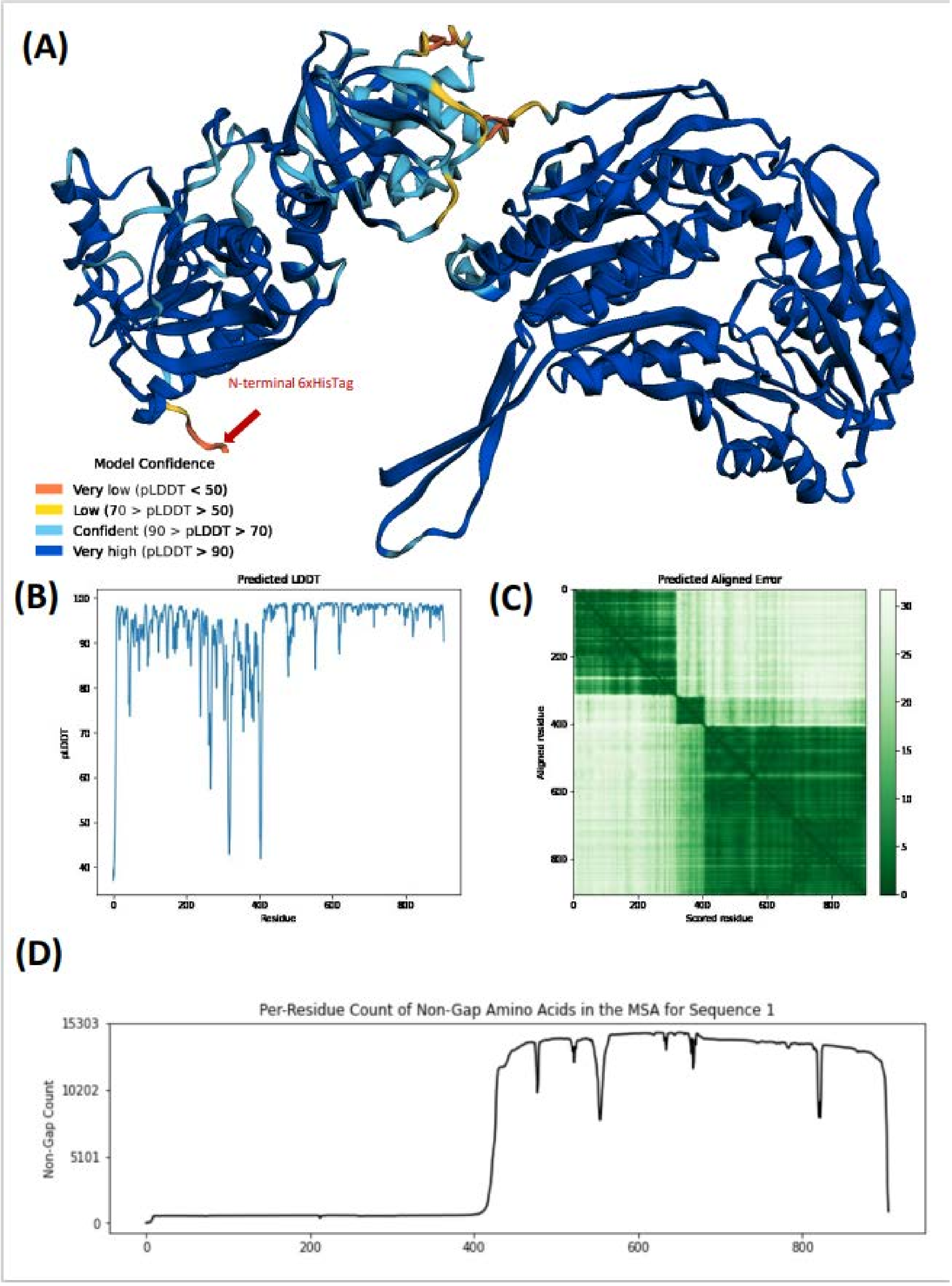

Figure S3 This figure shows the results of the Alphafold2 algorhytm. (A) shows the predicted model with the colors of the residues indicating the model confidence. Marled with a red arrow is the N-terminal 6xHisTag used for purification. (B) shows the predicted LDDT for each residue, where a high pLDDT indicates a high confidence of correct modeling of the repsective residue. (C) shows the predicted aligned error, which indicates the goodness of fit. (D) shows the results of an alphafold internal multiple sequence alignment (MSA) and how well each residue is covered by the MSA.

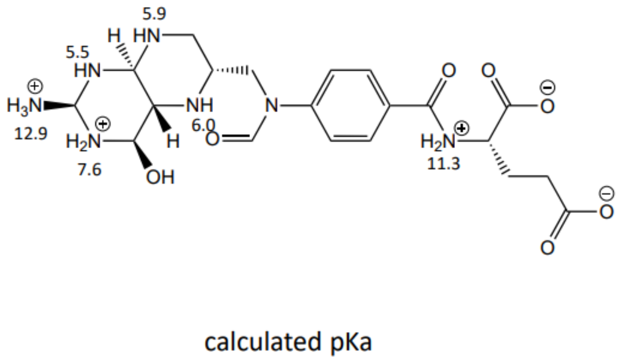

Figure S4 Calculated pKa values for the substrate 10-formyl-THF.

Table S1. List of Accession numbers for the 10-formyl-THFDH of used amino acid sequences.

species	Accession number
Apis cerana	XP_016904119.1
Apis dorsata	XP_031364038.1
Apis florea	XP_012342561.1
Apis laboriosa	XP_043797211.1
Apis mellifera	XP_006563851.2
Bombus bifarius	XP_033316336.1
Bombus huntii	XP_050492593.1
Bombus impatiens	XP_033176501.1
Bombus impatiens	XP_012240685.1
Bombus pyrosoma	XP_043602331.1
Bombus terrestris	XP_003403088.1
Bombus vancouverensis nearcticus	XP_033198288.1
Bombus vosnesenskii	XP_033364829.1
Eufriesea mexicana	XP_017754467.1
Homo sapiens	Uniprot_ O75891
Rattus norvegicus	Uniprot_ P28037
Mus musculus	Uniprot_ Q8R0Y6
Pongo abelii	Uniprot_ Q5RFM9
